# Hyperosmolarity and Increased Serum Sodium Concentration Are Risks for Developing Hypertension Regardless of Salt Intake: A Five-Year Cohort Study in Japan †

**DOI:** 10.3390/nu12051422

**Published:** 2020-05-14

**Authors:** Masanari Kuwabara, Mehmet Kanbay, Koichiro Niwa, Ryusuke Ae, Ana Andres-Hernando, Carlos A Roncal-Jimenez, Gabriela Garcia, Laura Gabriela Sánchez-Lozada, Bernardo Rodriguez-Iturbe, Ichiro Hisatome, Miguel A Lanaspa, Richard J Johnson

**Affiliations:** 1 Intensive Care Unit and Department of Cardiology, Toranomon Hospital, Tokyo 105-8470, Japan; 2 Cardiovascular Center, St. Luke’s International Hospital, Tokyo 104-8560, Japan; kniwa@aol.com; 3 Division of Renal Diseases and Hypertension, School of Medicine, University of Colorado Denver, Aurora, CO 80045, USA; ana.andreshernando@ucdenver.edu (A.A.-H.); carlos.roncal@ucdenver.edu (C.A.R.-J.); gabriela.garcia@ucdenver.edu (G.G.); miguel.lanaspagarcia@ucdenver.edu (M.A.L.); richard.johnson@ucdenver.edu (R.J.J.); 4 Division of Nephrology, Department of Internal Medicine, Koç University School of Medicine, 34010 Istanbul, Turkey; mkanbay@ku.edu.tr; 5 Division of Public Health, Center for Community Medicine, Jichi Medical University, Shimotsuke 329-0498, Japan; shirouae@jichi.ac.jp; 6 Department of Cardo-Renal Physiopathology, Instituto Nacional de Cardiología Ignacio Chávez, Mexico City 14080, Mexico; lgsanchezlozada@gmail.com; 7 Department of Nephrology, Instituto Nacional de Ciencias Medicas y Nutricion Salvador Zubiran and Instituto Nacional de Cardiologia Ignacio Chavez, Mexico City 14080, Mexico; brodrigueziturbe@gmail.com; 8 Division of Regenerative Medicine and Therapeutics, Tottori University Graduate School of Medical Sciences, Yonago 683-8503, Japan; hisatome@med.tottori-u.ac.jp

**Keywords:** osmolarity, hypertension, sodium, salt intake, epidemiology

## Abstract

The potential contribution of serum osmolarity in the modulation of blood pressure has not been evaluated. This study was done to examine the relationship between hyperosmolarity and hypertension in a five-year longitudinal design. We enrolled 10,157 normotensive subjects without diabetes who developed hypertension subsequently as determined by annual medical examination in St. Luke’s International Hospital, Tokyo, between 2004 and 2009. High salt intake was defined as >12 g/day by a self-answered questionnaire and hyperosmolarity was defined as >293 mOsm/L serum osmolarity, calculated using serum sodium, fasting blood glucose, and blood urea nitrogen. Statistical analyses included adjustments for age, gender, body mass index, smoking, drinking alcohol, dyslipidemia, hyperuricemia, and chronic kidney disease. In the patients with normal osmolarity, the group with high salt intake had a higher cumulative incidence of hypertension than the group with normal salt intake (8.4% versus 6.7%, *p* = 0.023). In contrast, in the patients with high osmolarity, the cumulative incidence of hypertension was similar in the group with high salt intake and in the group with normal salt intake (13.1% versus 12.9%, *p* = 0.84). The patients with hyperosmolarity had a higher incidence of hypertension over five years compared to that of the normal osmolarity group (*p* < 0.001). After multiple adjustments, elevated osmolarity was an independent risk for developing hypertension (OR (odds ratio), 1.025; 95% CI (confidence interval), 1.006–1.044), regardless of the amount of salt intake. When analyzed in relation to each element of calculated osmolarity, serum sodium and fasting blood glucose were independent risks for developing hypertension. Our results suggest that hyperosmolarity is a risk for developing hypertension regardless of salt intake.

**Running title:** Osmolarity and hypertension

## 1. Introduction

Salt intake is higher in Japan than in western societies, and averages more than 11 g per day in Japan and approximately 10 g per day in western countries [1,2]. There is extensive evidence that high salt intake is associated with hypertension, obesity, diabetes, and cardiovascular disease [2,3]. However, salt intake is associated with calorie intake, and calorie intake is directly associated with obesity, diabetes, and hypertension. Therefore, it is difficult to assess the direct association between salt intake and blood pressure. Some studies showed that salt intake below 6 g is associated with increased cardiovascular disease and increased all-cause mortality [4,5]. Moreover, Wald, et al. reported that a low serum sodium is associated with increased mortality risk [6].

The mechanisms by which salt intake is related to hypertension and cardiovascular disease are complex [7]. There is a moderate association between salt intake and blood pressure in hypertensive subjects, but a weak one for systolic blood pressure and salt intake and no association between salt intake and diastolic blood pressure in normotensive subjects [8,9]. A relative impairment in excretion of salt by the kidney appears to be a central mechanism, mediated by a local inflammatory reactivity and resulting in the interplay of vascular, cardiac, and sympathetic nervous system (SNS) responses [10,11]. The underlying mechanism by which the retention of salt causes the hypertensive response is complex and likely involves mechanisms beyond simple volume expansion. If the increase in osmolarity from salt intake is one of these mechanisms, then both the amount of salt and water ingested represent critically important variables in salt-sensitive hypertension.

Recently our group performed a clinical trial in which we administered a salty soup with or without water supplementation to human volunteers [12]. We observed an acute rise in blood pressure that correlated with the rise in serum osmolarity, and this was blocked by concomitant water intake that prevented the rise in osmolarity [12]. While these observations apply only to an acute sodium load, it led us to hypothesize that similar hypertensive effects of hyperosmolarity may be playing a role in the development of high blood pressure related to a chronic high-salt diet in the general population.

## 2. Materials and Methods

### 2.1. Study Design and Study Subjects

The five-year study was done in a single center and was designed to clarify the risk factors for developing hypertension. We used the database of the general subjects who underwent annual regular health check-up by themselves both in 2004 and 2009 at the Center for Preventive Medicine, St. Luke’s International Hospital, Tokyo, Japan. This data base and details of the study have been the subject of several previous publications [13,14,15,16,17,18,19,20,21]. In all, 10,157 normotensive subjects without diabetes mellitus, ranging in age from 30 to 85 years were included. We excluded subjects with hypertension because the study objective was to clarify risk factors for the new development of hypertension. We also excluded subjects with diabetes in order to minimize errors in the calculation of osmolarity resulting from blood glucose variability.

We analyzed the medical records, general history for comorbidities, and questionnaire of the study subjects. When the study subjects had examinations more than once a year, we used only the first results in the same year to avoid double counting of the data. The dietary history was based on a self-answered questionnaire by a single, 24 hour recall as reported in our previous study [3]. The dietary questionnaire has been used in prior epidemiological studies [22,23]. Every subject underwent physical and laboratory examinations. All blood samples were collected in the morning and performed in the same laboratory. However, the study design had some limitations, as it was a retrospective, single-center study. Serum osmolarity was calculated rather than directly measured, and the effects of medication except for antihypertensive or diabetic medicine were not evaluated. Salt intake was also determined by a questionnaire method, and the study did not assess antidiuretic hormone levels, the renin–angiotensin–aldosterone system, or overall water intake.

### 2.2. Definition of Hypertension, Diabetes, Dyslipidemia, Hyperuricemia, Chronic Kidney Disease, and Serum Osmolarity

Hypertension was defined as systolic blood pressure ≥140 mmHg and/or diastolic blood pressure ≥90 mmHg [24,25] and/or as present in the subjects who had medication for hypertension at the time. Blood pressure readings were obtained using an automatic brachial sphygmomanometer (OMRON healthcare Co., Ltd., Kyoto, Japan). Blood pressure determinations were taken after the participant had been seated and resting quietly for more than five minutes, with their feet on the ground and back supported. The means of two determinations each of systolic and diastolic blood pressure were used. Diabetes was considered to be present in subjects who had current history of diabetes mellitus and/or a glycosylated hemoglobin (HbA1c) (National Glycohemoglobin Standardization Program (NGSP)) value ≥6.5% [26]. Dyslipidemia was considered to be presen in subjects who were receiving treatment for dyslipidemia and/or had a low-density lipoprotein cholesterol level ≥140 mg/dL, a high-density lipoprotein cholesterol level <40 mg/dL, and/or a triglyceride level ≥150 mg/dL [27]. Hyperuricemia was defined by a serum uric acid level higher than 7.0 mg/dL, in accordance with Japanese guidelines [28]. Chronic kidney disease was defined by an estimated glomerular filtration rate (eGFR) <60 mL/min/1.73 m^2^, calculated by the Japanese GFR equation (eGFR (mL/min/1.73 m^2^) = 194 × Serum creatinine^−1.094^ × age^−0.287^ (×0.739 if women)) [29]. The serum creatinine was measured by enzymatic analysis and calibrated to the information display measurements standard (IDMS). Serum osmolarity (mOsm/L) was calculated using a formula that takes into account serum sodium, blood urea nitrogen (BUN), and blood glucose; (2 × Sodium) + (BUN/2.8) + (Glucose/18) [30]. Alcohol drinking habits were defined as positive if the individual drank daily, and no alcohol consumption or only social drinking was considered negative. A positive smoking history included both current and past use of smoking tobacco.

### 2.3. The Cutoff Point between Hyperosmolarity and Normal Osmolarity and between High Salt Intake and Normal Salt Intake

Hyperosmolarity was defined as serum osmolarity higher that 293 mOsm/L (the mean value of the study subjects). High salt intake was defined as dietary salt ingestion higher that 12 g per day (the mean value of the study subjects). Serum osmolarity values were the mean levels of the study subjects, and the value of salt intake was similar to the mean salt intake in the Japanese population [1]. The cumulative incidence of hypertension over 5 years was evaluated in the groups with high or normal serum osmolarity and with high or normal salt intake.

### 2.4. Statistical Analysis

All statistical analyses were performed using the SPSS Statistics software (IBM SPSS Statistics version 22 for Windows; IBM, New York). The statistically significant level was set at α = 0.05. All of the statistical analyses were two-sided. Bivariate associations between demographic and clinical characteristics were compared between men and women using *t*-tests and χ^2^ analyses. The analyses among serum osmolarity quartile, salt intake quartile, or in each serum sodium for cumulative incidence of hypertension over five years were conducted by the Mantel–Haenszel test for trend. The risk factors for the development of hypertension were evaluated by logistic regression analyses with multiple adjustments for age, gender, body mass index, smoking, drinking alcohol, dyslipidemia, hyperuricemia, chronic kidney disease, and serum osmolarity. In addition, we conducted logistic regression analyses for the element of the equation for the calculation of serum osmolarity: serum sodium, blood glucose, and BUN.

### 2.5. Ethical Considerations

We adhered to the principles of the Declaration of Helsinki. All data were collected and compiled in a protected computer database. Individual data were anonymized, and there was no personality information identified. St. Luke’s International Hospital Ethics Committee approved the protocol for this study (Ethical approval ID, 16-R025). We had consent from all the subjects by a comprehensive agreement (opt-out) method provided by the hospital.

## 3. Results

### 3.1. Characteristics of Study Subjects

**Figure 1:**
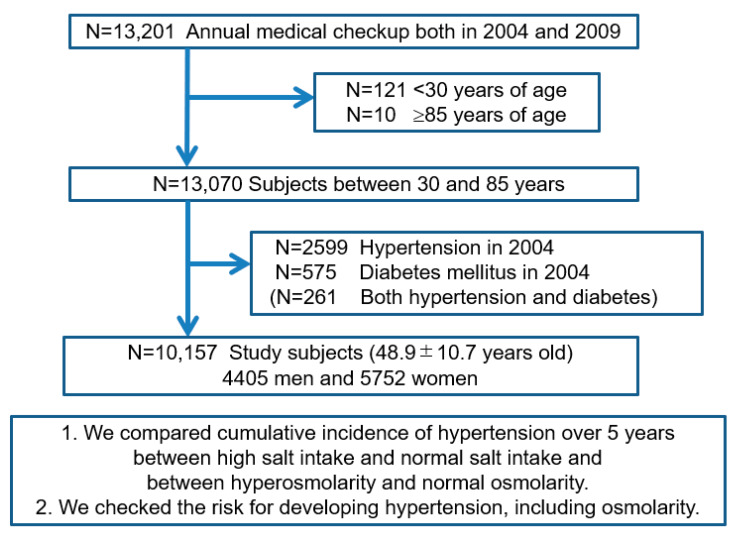
Flow diagram of study enrollment. Of 13,201 subjects who underwent annual medical examinations at the center in 2004 and again in 2009, we enrolled 10,157 subjects (4405 men) between 30 and 85 years old without hypertension and diabetes mellitus at the baseline (in 2004).

We retrospectively analyzed the medical records of 13,201 subjects who underwent annual medical examinations at the center both in 2004 and 2009. Of those, 13,070 subjects were within the predetermined age range of 30–85 years of age at baseline. We excluded 2599 subjects with hypertension, 575 subjects with diabetes, and 261 who had both hypertension and diabetes. Ultimately, 10,157 subjects (4405 men and 5752 women) were enrolled to identify risk factors for developing hypertension over a five-year period (Figure 1). Baseline demographic data on the study subjects are shown in Table 1.

### 3.2. Cumulative Incidence of Hypertension over Five Years between Hyperosmolarity and Normal Osmolarity and between High and Normal Salt Intake

The cumulative incidence of hypertension over five years was 10.2% (1040/10,157), 13.5% (594/4405) in men, and 7.8% (446/5752) in women. Table 1 shows the baseline data for the group that developed hypertension compared to the group that remained normotensive. The group that developed hypertension was significantly older and more frequently male. They had a higher body mass index, higher rate of smoking, higher incidence of drinking alcohol, worse dyslipidemia, with more hyperuricemia, chronic kidney disease, lower eGFR, higher fasting blood glucose, higher BUN, higher serum sodium, higher salt intake, and higher serum osmolarity.

We also evaluated the risk for hypertension in those with baseline hyperosmolarity compared with the normal osmolarity group, and the risk was significantly greater in the hyperosmolar group (13.0% versus 7.5%, *p* < 0.001). The high salt intake group also was associated with a significantly higher cumulative incidence of hypertension compared to the normal salt intake group (10.9% versus 9.7%, *p* = 0.046), but the difference was smaller.

**Figure 2:**
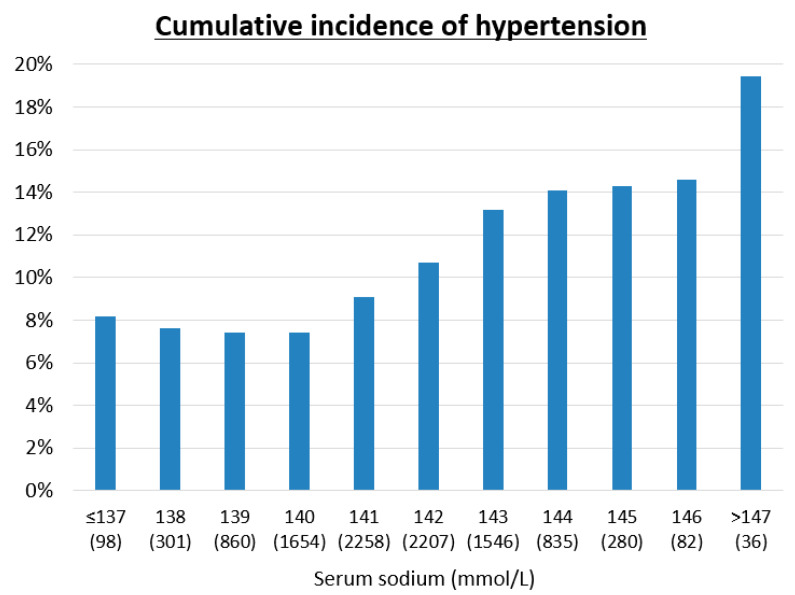
Cumulative incidence of hypertension over five years in each serum sodium level. The analysis among each serum sodium was conducted by Mantel-Haenszel test for trend (*p* = 0.001).

We also checked the cumulative incidence of hypertension over five years among quartiles of serum osmolarity (Table 2(A)), among quartiles of salt intake (Table 2(B)), and for each serum sodium level, all measured at baseline (Figure 2). The group with higher serum osmolarity (Table 2(A)) or higher salt intake (Table 2(B)) had a significantly higher cumulative incidence of hypertension by the Mantel–Haenszel test for trend (*p* < 0.001). Moreover, higher serum sodium also had a significantly higher cumulative incidence of hypertension by the Mantel–Haenszel test for trend (*p* < 0.001) (Figure 2).

**Figure 3:**
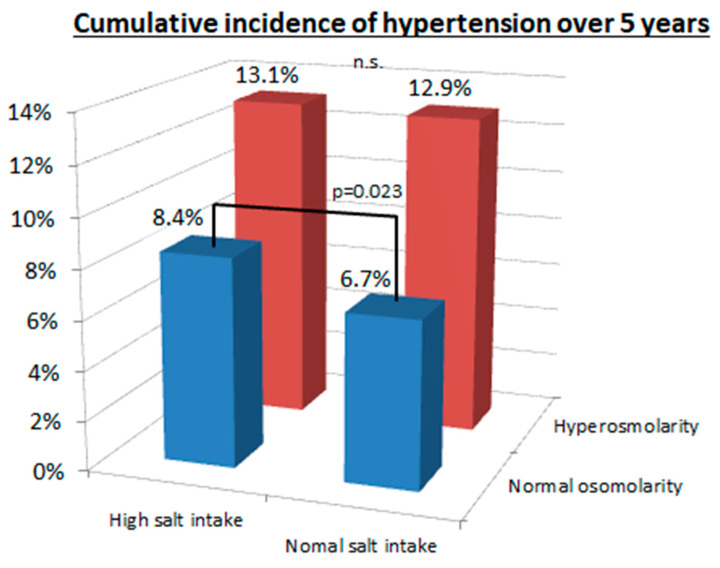
Cumulative incidence of hypertension between hyperosmolarity and normal osmolarity and between high and normal salt intake. There was a significant difference in cumulative incidence of hypertension between high salt intake (>12 g/day) and normal salt intake (≤12 g/day) in the normal osmolarity group (8.4% versus 6.7%, *p* = 0.023), but not in the hyperosmolarity group (13.1% versus 12.9%, *p* = 0.84). In contrast, the hyperosmolarity group (>293.3 mOsm/L) had significantly higher cumulative incidence of hypertension compared with the normal osmolarity group (≤293.3 mOsm/L) both in the high salt intake group (13.1% versus 8.4%, *p* < 0.001) and the normal salt intake group (12.9% versus 6.7%, *p* < 0.001).

The relationship of serum osmolarity with salt intake is shown in Figure 3. The group on a high salt intake had a higher cumulative incidence of hypertension compared to the normal salt intake group in patients with normal osmolarity (8.4% versus 6.7%, *p* = 0.023), but not in patients with hyperosmolarity (13.1% versus 12.9%, *p* = 0.84). In contrast, the patients with hyperosmolarity had a higher cumulative incidence of hypertension than patients in the normal osmolarity group (≤293 mOsm/L) regardless of whether they were on a high salt intake (13.1% versus 8.4%, *p* < 0.001) or normal salt intake (12.9% versus 6.7%, *p* < 0.001).

### 3.3. Risk Factors for Developing Hypertension

After multiple adjustments with age, gender, body mass index, smoking, drinking alcohol, dyslipidemia, hyperuricemia, chronic kidney disease, and salt intake, higher serum osmolarity was an independent risk factor for developing hypertension (OR (odds ratio), 1.025; 95% CI (confidence interval), 1.006–1.044; *p* = 0.010), as well as aging, higher body mass index, drinking alcohol, and hyperuricemia (Table 3(A)). When conducting similar analyses as done for calculated serum osmolarity, higher serum sodium (OR, 1.045; 95% CI, 1.005–1.087; *p* = 0.028) and higher fasting blood glucose (OR, 1.018; 95% CI, 1.010–1.026, *p* < 0.001) both were independent risk factors for developing hypertension, whereas BUN was not (*p* = 0.90) (Table 3(B)).

We conducted an additional sensitivity analysis with baseline systolic blood pressure. The results showed that baseline blood pressure was an independent risk for developing hypertension (OR per 1 mmHg increase, 1.122; 95% CI, 1.113–1.131), and, when correcting for baseline blood pressure, that calculated serum osmolarity was no longer significant (OR per 1 mOsm/L increase, 1.084; 95% CI, 0.929–1.264).

## 4. Discussion

The primary finding in our study is that serum osmolarity, and especially serum sodium concentration, could predict the development of hypertension while sodium intake itself was only a weak predictor. The increased risk was independent of multiple other well-known risk factors, including age, gender, body mass index, dyslipidemia, chronic kidney disease, and hyperuricemia. There was a 13% increased risk of hypertension for every 5 mOsm/L increment in serum osmolarity. Specifically, for every 1 mmol/L rise in serum sodium the risk for hypertension was increased by 4.5%, and for those with serum sodium >144 mmol/L, the cumulative incidence of hypertension over five years was 14.8% compared to 7.5% in those with a serum sodium of <140 mmol/L. These findings suggest that the balance of salt and water intake is the most important dietary factor in the development of hypertension.

Recent studies have raised the potential importance of hyperosmolarity as a driver of hypertension [31,32,33]. An increase in serum osmolarity by salt has been shown to activate the aldose reductase–fructokinase pathway in the liver and hypothalamus that can over time lead to metabolic syndrome and elevations in blood pressure [3]. The increase in serum and SNS osmolarity stimulates vasopressin and leptin release [34], and the latter can activate the SNS sympathetic response to raise blood pressure [35]. An increase in SNS osmolarity also stimulates the production of cardiotonic steroids (Na–K ATPase) inhibitors that cause peripheral vasoconstriction [36]. Hyperosmolarity also activates both macrophages and T cells in the immune system that may enhance the inflammatory responses that cause persistent renal vasoconstriction [37,38].

In our study, we did not find a strong relationship of salt intake with the development of hypertension. Other studies have also occasionally found weak relationships, such as the original analysis of the Intersalt Study (discussed in [39]). Kitada et al. reported that high salt intake reprioritizes osmolyte and energy metabolism for body fluid conservation regulated by concerted liver, muscle, and renal actions [40]. Indeed, we recommend future studies that address the overall relationship of water and salt intake in the hypertensive response. Furthermore, one might posit that the reason the thiazide diuretics are more effective at lowering blood pressure than loop diuretics is not due to the sodium loss, as loop diuretics are more potent in inducing salt excretion, but rather because thiazides tend to lower serum sodium while loop diuretics have the opposite action.

This study also showed that high fasting blood glucose concentration is an independent risk factor for developing hypertension. Some recent studies also support our results, that fasting blood glucose is independently associated with the new development of hypertension [41,42]. We have also showed the positive association of blood glucose levels and hypertension even when blood glucose is in the normal range, less than 120 mg/dL [18]. As summarized previously [43], higher blood glucose increases advanced glycation end products, inflammation, oxidative stress, and vascular dysfunction [44,45], which could contribute to the development of hypertension. In addition, blood glucose is also an element of serum osmolarity, which could represent an additional factor to play a role in hypertension.

This study has several limitations. First, this study was a retrospective, single-center study, which may introduce selection bias. However, there are some merits of a single-center study including that all subjects had identical laboratory tests performed with the same laboratory. Second, serum osmolarity was calculated rather than directly measured. However, an advantage is that we could analyze individually each element of calculated serum osmolarity and found that serum concentrations of sodium and fasting glucose are also independent risk factors for developing hypertension. Third, we could not include effects of medication in the study. However, subjects with any medication for hypertension and/or diabetes at baseline were excluded from the study, which minimized the limitations on the lack of medication information. Fourth, the amount of salt intake was measured by a questionnaire method, which may not be completely accurate. We recognize that direct measurement is the best method, but this is impossible in a retrospective study. Nevertheless, the questionnaire in this study has been used before [22,23,46] and found useful. Fifth, our study could not assess antidiuretic hormone, renin–angiotensin system, aldosterone, and water intake due to being a retrospective study. These factors are important to assess serum sodium and osmolarity. Finally, our study could not show the causality because of the nature of observational studies.

Another issue is that the baseline blood pressure in the group that developed hypertension was higher than the baseline blood pressure in the non-hypertensive group. Therefore, we conducted additional sensitivity analysis with baseline systolic blood pressure. The results showed that baseline blood pressure was an independent risk for developing hypertension, and, when correcting for baseline blood pressure, that calculated serum osmolarity was no longer significant. One reason could be that higher serum osmolality was associated with higher blood pressures at baseline, and so the two factors may be difficult to separate. Nevertheless, this issue is a limitation of this study. Further studies are needed to clarify the potential benefit of controlling serum osmolarity to prevent the development of hypertension.

## 5. Conclusions

In conclusion, our study highlights the importance of osmolarity in driving blood pressure response. We continue to support guidelines to restrict sodium intake as a key preventive measure for improving cardiometabolic health. However, we suggest that further studies should evaluate the balance of salt and water intake as major determinants of serum osmolarity, as we would predict this will have a greater impact than only focusing on salt.

## Figures and Tables

**Table 1:** Baseline demographic data on the study subjects and the difference of baseline data between the developing hypertension (HT) group and non-hypertension (non-HT) group.

		Total	Non-HT	HT	*p*
Number of subjects		10,157	9117	1040	
Age	years old	48.9 ± 10.7	48.3 ± 10.5	54.3 ± 10.7	<0.001
Male	%	43.4	41.8	57.1	<0.001
Body mass index	kg/m^2^	22.0 ± 2.9	21.8 ± 2.9	23.4 ± 3.2	<0.001
Smoking	%	36.3	35.5	40.8	<0.001
Drinking alcohol	%	41.5	40.8	48.1	<0.001
Dyslipidemia	%	34.6	33.2	47.2	<0.001
Hyperuricemia	%	11.8	10.8	19.9	<0.001
Chronic kidney disease	%	2.5	2.4	3.8	0.017
Systolic blood pressure	mmHg	113.0 ± 13.1	111.4 ±12.5	127.2 ± 8.7	<0.001
Diastolic blood pressure	mmHg	70.5 ± 8.8	69.4 ± 8.4	79.8 ± 6.3	<0.001
Fasting blood glucose	mg/dL	96.9 ± 8.6	96.5 ± 8.4	100.4 ± 9.3	<0.001
Blood urea nitrogen	mg/dL	13.8 ± 3.3	13.7 ± 3.3	14.4 ± 3.6	<0.001
Serum sodium	mmol/L	141.5 ± 1.8	141.4 ± 1.7	141.9 ± 1.8	<0.001
Serum potassium	mmol/L	4.18 ± 0.28	4.18 ± 0.28	4.21 ± 0.29	<0.001
Serum osmolarity	mOsmol/L	293.3 ± 4.0	293.1 ± 3.9	294.5 ± 3.9	<0.001
Estimated GFR	mL/min/1.73 m^2^	86.7 ± 15.3	87.1 ± 15.3	83.5 ± 15.1	<0.001
Salt intake	g/day	12.2 ± 3.7	12.2 ± 3.6	12.6 ± 3.9	0.046

HT, hypertension; GFR, glomerular filtration rate; mOsm/L, osmolarity per liter; bpm, beats per minute; *p*, probability. Values are expressed as mean ± standard deviation. Serum osmolarity (mOsm/L) was calculated using a formula that takes into account serum sodium, blood urea nitrogen (BUN), and blood glucose: (2 × Sodium) + (BUN/2.8) + (Glucose/18) [30].

**Table 2:** Cumulative incidence of hypertension over five years among quartiles of serum osmolarity (**A**) and among quartiles of salt intake (**B**).

**(A)**					
**Osmolarity (mOsmol/L)**	**≤290**	**290–293**	**293–296**	**>296**	***P***
Number of subjects	2128	2663	2873	2493	
Cumulative incidence	6.3%	8.4%	10.9%	14.8%	<0.001
**(B)**					
**Salt intake (g/day)**	**≤10**	**10–12**	**12–14**	**>14**	***P***
Number of subjects	2925	2320	2061	2842	
Cumulative incidence	9.3%	10.1%	9.7%	11.7%	0.006

(**A**) The analysis among each serum osmolarity quartile was conducted by the Mantel–Haenszel test for trend (*p* < 0.001). The number of subjects was 2128 in the 1st quartile (≤290 mOsm/L), 2663 in the 2nd quartile (290–293 mOsm/L), 2873 in the 3rd quartile (293–296 mOsm/L, and 2493 in the 4th quartile (>296 mOsm/L) of serum osmolarity. (**B**) The analysis among each salt intake quartile was conducted by the Mantel–Haenszel test for trend (*p* = 0.006). The number of subjects was 2925 in the 1st quartile (≤10 g/day), 2320 in the 2nd quartile (10–12 g/day), 2061 in the 3rd quartile (12–14 g/day), and 2842 in the 4th quartile (>14 g/day) of salt intake.

**Table 3:** Risk factors for hypertension over five years after multiple adjustments using serum osmolarity (**A**) and each element of serum osmolarity, namely, blood urea nitrogen, serum sodium, and fasting blood glucose (**B**).

**(A)**		**OR**	**95% CI**	***p***
Age	per 1 year increase	1.052	1.045–1.059	<0.001
Men	versus women	1.006	0.847–1.195	0.95
Body mass index	per 1 kg/m^2^ increase	1.170	1.145–1.203	<0.001
Smoking habits	versus negative	1.012	0.867–1.182	0.88
Drinking alcohol	versus negative	1.297	1.120–1.502	<0.001
Dyslipidemia	versus negative	1.047	0.909–1.206	0.52
Hyperuricemia	versus negative	1.295	1.070–1.568	0.008
Chronic kidney disease	versus negative	0.710	0.493–1.023	0.71
Serum osmolarity	per 1 mOsm/L increase	1.025	1.006–1.044	0.010
Salt intake	per 1 g/day increase	0.996	0.979–1.014	0.66
**(B)**		**OR**	**95% CI**	***p***
Age	per 1 year increase	1.051	1.043–1.058	<0.001
Gender (Male)	versus women	0.965	0.809–1.151	0.69
Body mass index	per 1 kg/m^2^ increase	1.162	1.133–1.191	<0.001
Smoking habits	versus negative	1.001	0.857–1.170	0.99
Drinking alcohol	versus negative	1.248	1.076–1.447	0.003
Dyslipidemia	versus negative	1.030	0.894–1.187	0.68
Hyperuricemia	versus negative	1.290	1.065–1.562	0.009
Chronic kidney disease	versus negative	0.743	0.514–1.073	0.113
Blood urea nitrogen	per 1 mg/dL increased	0.999	0.977–1.021	0.90
Serum sodium	per 1 mmol/L increased	1.045	1.005–1.087	0.028
Fasting blood glucose	per 1 mg/dL increased	1.018	1.010–1.026	<0.001
Salt intake	per 1 g/day increased	0.996	0.978–1.014	0.66

OR: odds ratio, CI: confidence interval, p: probability. (**A**) Analysis with serum osmolarity: Data were adjusted for age, gender, body mass index, smoking, drinking alcohol, dyslipidemia, hyperuricemia, chronic kidney disease, serum osmolarity, and salt intake. Serum osmolarity (mOsm/L) was calculated using a formula that takes into account serum sodium, blood urea nitrogen (BUN), and blood glucose: (2 × Sodium) + (BUN/2.8) + (Glucose/18) [30]. (**B**) Analysis with blood urea nitrogen (BUN), serum sodium, and fasting blood glucose, instead of calculated serum osmolarity: Data were adjusted for age, gender, body mass index, smoking, drinking alcohol, dyslipidemia, hyperuricemia, chronic kidney disease, blood urea nitrogen, serum sodium, fasting blood glucose, and salt intake.
